# Review projecting shikonin as a therapeutic candidate in female carcinomas: a preclinical perspective

**DOI:** 10.3389/fphar.2025.1627124

**Published:** 2025-07-04

**Authors:** Pratibha Pandey, Sorabh Lakhanpal, K. V. Jamuna, Ajay Singh, Mohammad Abohassan, Moon Nyeo Park, Sang-Won Shin, Han Na Kang, Manaal Zahera, Mohd Saeed, Fahad Khan, Bonglee Kim

**Affiliations:** ^1^ Research and Innovation Cell, Rayat Bahra University, Mohali, Punjab, India; ^2^ School of Pharmaceutical Sciences, Lovely Professional University, Phagwara, Punjab, India; ^3^ Department of Forensic Science, School of Sciences, JAIN (Deemed to be University), Bangalore, Karnataka, India; ^4^ School of Applied and Life Sciences, Uttaranchal University, Dehradun, India; ^5^ Department of Clinical Laboratory Sciences, College of Applied Medical Sciences, King Khalid University, Abha, Saudi Arabia; ^6^ Health and Medical Researches Centre, King Khalid University, Abha, Saudi Arabia; ^7^ Department of Pathology, College of Korean Medicine, Kyung Hee University, Seoul, Republic of Korea; ^8^ Department of Humanities and Social Medicine, School of Korean Medicine, Pusan National University, Yangsan-si, Republic of Korea; ^9^ KM Convergence Research Division, Korea Institute of Oriental Medicine, Daejeon, Republic of Korea; ^10^ Department of Biotechnology, Era University, Lucknow, Uttar Pradesh, India; ^11^ Department of Biology, College of Science, University of Hail, Hail, Saudi Arabia; ^12^ Center for Global Health Research, Saveetha Medical College, Saveetha Institute of Medical and Technical Sciences, Chennai, Tamil Nadu, India

**Keywords:** shikonin, female cancer, anticancer, phytochemical, naphthoquinone

## Abstract

Bioactive substances, especially shikonin (naphthoquinone), which is extracted from Lithospermum erythrorhizon, have drawn much attention as promising substitutes for preventing cancer malignancy. Shikonin (SK) has displayed a broad spectrum of anticancer activities, such as necroptosis, cell cycle invasion, Autophagy, apoptosis, Diabetes, DNA damage induction, and suppression of angiogenesis. It reverses drug resistance and inhibited cancer cell growth by altering their metabolism. According to preliminary clinical trials, shikonin may improve the effectiveness of known chemotherapeutic drugs, radiation therapies, and immunotherapies through synergistic and additive interactions in female carcinomas. Despite its potential, additional investigation is required to pinpoint exact processes by which shikonin causes metabolic reprogramming in female cancers. While numerous researches have been reported to understanding the anticancer potential of shikonin, more research is needed to investigate its synergistic effects with conventional cancer therapies and assessing its clinical efficacy in robust trials. Due to less clinical data, more number of clinical trials is vital to establish their efficacy and safety in human patients, while mechanistic experimentation could unveil new therapeutic oncotargets in managing female carcinomas.

## 1 Introduction

Shikonin (SK), a traditional Chinese herbal medicine (red naphthoquinone compound) extracted from the dried roots of *Lithospermum erythrorhizon*. SK and its derivatives exhibit various pharmacological effects, such as anti-inflammatory properties, regulation of oxidative balance, and immune system monitoring (Biswal and Biswal, 2024). Recent *in vitro* studies have shown that SK has potent anti-tumor effects in lung cancer, colon cancer, and other tumors (Ma et al., 2021). Mechanistically, shikonin induces cell death irrespective of p53 status, inhibited ERK-dependent cell growth signals, and halts cell cycle (at G2/M phase). Collectively, these actions contribute to the growth-inhibitory effects of shikonin (Chen et al., 2002). SK induced cell death was significantly reduced *via* pan-apoptosis inhibitor, highlighting the importance of apoptosis in this phenomenon (Han et al., 2007). Interestingly, Shikonin further initiated autophagy and the protective role of autophagy was evidenced *via* increased cell death resulting from the suppression of autophagy through the depletion of essential autophagic genes. To elucidate the underlying mode of action behind these effects, shikonin was shown to upregulate p21, autophagy genes and suppression of the genes vital for cell development (Xu et al., 2022). SK has projected a broader range of bioactivities, including anti-inflammatory, wound healing, anti-HIV, and anticancer potential (Chen et al., 2003). It has been broadly considered as an anticancer agent, showing promising results (both *in vitro* and *vivo*), as it appears to cause minimal harm to healthy tissues and organs (Wang et al., 2020). The roots of Zicao contain shikonin, a natural red compound with a naphthoquinone structure (Bichave et al., 2024). Shikonin activates signaling pathways that regulated cytoskeleton formation, mitochondrial dysfunction, and oxidative stress responses. When this compound gets accumulated in mitochondria, it produced ROS and disrupted intracellular Ca^2+^ levels. Thus, cell cycle (cell growth) arrest and apoptosis occur due to microtubules disruption and mitochondrial membrane potential. Therefore, shikonin could serve as a parent compound for developing new gynecological anticancer drugs (Ke et al., 2022). The core parent nuclear structure of shikonin is 5, 8-dihydroxy-1, 4-naphthoquinone with isohexenyl side chains (Shukla et al., 2021). SK compounds further classified into two optical isomers, S and R shikonin (based on their optical activities) (Braun and Bauer 1991). Moreover, the anti-cancer effects of SK are more significant and widespread than those of alkannin. This study elucidated the anti-gynecological potential of SK to broaden its application in gynecological diseases. This study highlighted it as a powerful anti-female cancer therapy candidate by utilizing a variety of databases, such as Web of Science, Scopus, Google Scholar, and PubMed.

## 2 Shikonin and its pharmacokinetics

Shikonin is a type of 1,4-naphthoquinone (1,4-NQ) with C6–C4 skeleton structure. These compounds are secondary metabolites found in plants, fungi, and microorganisms. Shikonin (deep red) pigment primarily found in L. erythrorhizon roots can be easily extracted (Yazaki, 2017). Researchers have extensively studied shikonin and its both (natural or synthetic) derivatives to develop compounds with enhanced pharmacological properties, such as improved target specificity, increased water solubility, and reduced toxicity to normal tissues (Andújar et al., 2013). Due to shikonin’s high toxicity and low solubility, which limit its use as an anticancer drug, the concept of combinatorial medications has been applied. For instance, α-lipoic acid (cofactor of pyruvate dehydrogenase) has been combined with SK treatment. Eighteen ester derivatives of shikonin hybrids and α-lipoic acid were tested against various cancer cells. Only one compound showed significant PDK1 inhibition and notable cytotoxicity against HeLa cells. This derivative’s enhanced aerobic metabolism led to apoptosis induction, tubulin polymerizationprevention, and G2/M cell cycle arrest (Lin H. Y. et al., 2018). Naphthoquinones substituted with 2, 3-dithiocarbamate was evaluated for their ability to inhibit M2 isoform of pyruvate kinase (PKM2).

Two derivatives exhibited superior PKM2 inhibitory activity compared to shikonin. Most compounds demonstrated IC_50_ in the nanomolar range when tested against HCT116, B16, MCF7, HeLa, and H1299 cells (Idris et al., 2024). Shikonin coumarin carboxylic acid is another noteworthy compound due to its ability of apoptotic induction by inhibiting HIF-1α expression in HeLa cells (Mishra et al., 2024). SK derivative (β-HIVS) treated HeLa cells displayed reduced expression levels of mTOR, S6 kinases (70-kDa ribosomal protein), AKT, and PI3K were reduced, and cell cycle was arrested in the S phase. Additionally, inhibition of PI3K/AKT/mTOR cell signaling pathway was responsible for inducing apoptosis (Xu et al., 2022). Detailed pharmacokinetics and toxicology of shikonin has been reported in several studies hence we have not included specific section (Yadav et al., 2022; Cui et al., 2025; Olatunde et al., 2024)

## 3 Effects of shikonin on female malignant carcinoma

Cervical, endometrial, ovarian, and breast carcinomas are widespread malignant carcinomas in females (Kaveh et al., 2016). Additionally, the incidence and mortality of female malignancies have increased recently, putting women’s lives and health in grave danger. These days, surgery, chemotherapy, and radiation therapy are the primary therapeutic modalities utilized for female malignant tumors. Of these, chemotherapy is particularly useful in the treatment of gynecological malignancies (Wright et al., 2016). As a result, we summarized how shikonin affected all prevailing malignant tumors in women.

### 3.1 Anti-cancer potential of shikonin against cervical carcinoma

**FIGURE 1:**
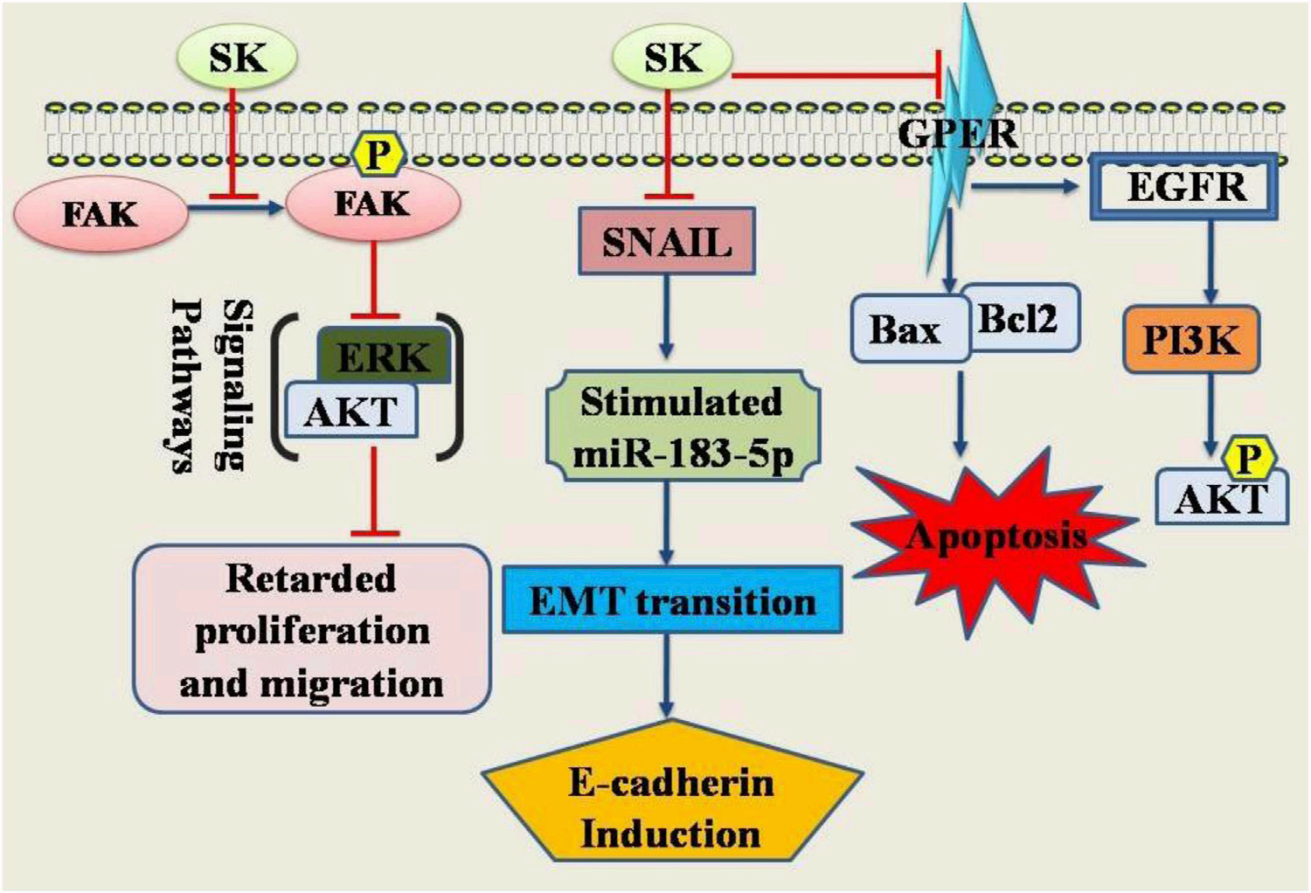
Diagrammatic illustration of the anti-cervical cancer potential of shikonin. Shikonin has been shown its inhibitory or promoting effects on certain genes or proteins required either for the cancer cell death or inhibition of the genes required for apoptosis in cancer cells. Abbreviations: ERK, Extracellular Signal-Regulated Kinase; SNAIL, Snail family transcriptional repressor 1; FAK, Focal Adhesion Kinase; EGFR, Epidermal growth factor receptor; PI3K, Phosphoinositide 3-kinase.

Shikonin, the main component of *L. erythrorhizon*, has demonstrated anticancer properties by affecting various targets and signaling pathways. It suppresses EMT by downregulating Snail and upregulating miR-183-5p, leading to increased E-cadherin expression. This suggests that shikonin’s anti-cervical cancer effects might stem from its unique ability to inhibit EMT (Tang et al., 2020). Additionally, shikonin targets the dephosphorylation of Cdc25s and CDCK1, thereby halting the cell cycle in cancerous cells. These findings indicate that shikonin can inhibit Cdc25s, causing cellular growth arrest in (both *in vitro* and *vivo*) (Zhang et al., 2019). Furthermore, shikonin exhibits significant anti-proliferative effects on HeLa and SiHa cervical cells *via* inhibiting FAK/AKT/GSK3β signaling pathway (Shukla et al., 2021). It has been shown to exert anticancer effects through multiple signaling pathways and targets. Shikonin reduces the expression of vimentin and snail while enhancing miR-183-5p expression and E-cadherin protein expression and promoter activity (Tang et al., 2020) (Figure 1).

Significant attention has been devoted to prior studies focused on the synthesis or extraction of high-performance, non-toxic shikonin derivatives. One such naturally occurring derivative is β-hydroxyisovaleryl-shikonin (β-HIVS) (Lu et al., 2013; Rajasekar et al., 2012). This compound induces apoptosis in HeLa cells *via* altering PI3K/AKT/mTOR signaling (in dose-dependent manner) (Lu et al., 2015). An additional biological component derived from *Lithospermum erythrorhizon* is deoxyshikonin. Given that the anticancer efficacies of deoxyshikonin on HeLa cancer cells are not well understood, further research was undertaken to explore its anticancer efficacy and the underlying molecular mechanisms. Findings from an apoptosis microarray, which aimed to elucidate the relevant pathways, revealed that DSK reduced cellular inhibitor of apoptosis proteins (cIAP1, cIAP2 and XIAP) (in dose-dependent manner) and triggered PARP cleavage and caspase-8/9/3 activation (Lee et al., 2024). Deoxyshikonin induced apoptosis in HeLa cells *via* p38-mediated caspase activation and IAP downregulation.

Acetylshikonin (naphthoquinone), derived from Chinese herb (dried purple roots) L. erythrorhizon Sieb. Et Zucc., possess several biological potential, including anti-HIV, antibacterial, antifungal, antipyretic, antimicrobial, anti-inflammatory, antitumor, and analgesic effects (Zhang et al., 2020). It has shown significant antiproliferative effects by halting cells from progressing from the S to the G2/M phase and apoptosis induction in SiHa cells *via* activating caspases (3 and 8). Consequently, acetylshikonin is considered a promising natural candidate for the efficient treatment of cervical carcinoma (Sun et al., 2014). Table 1 compiles all the significant research on the mechanism of action of shikonin against cervical cell cancer.

**TABLE 1:** Anti-cancer potential of shikonin against cervical cancer cells at different doses.

Cancer cell line (s)	Dose (s)	Mode of action	References
HeLa Cells	Shikonin (0, 1, 5 and 25 μM)	Induced irreversible inhibition of human recombinant CDC25 phosphatases Hyper-phosphorylation of CDKs Cell cycle arrest	Zhang et al. (2019)
tsFT210 (temperature-sensitive FT210 mouse breast cancer cell line)	Shikonin (0, 1, 5 and 25 μM)	Induced cell cycle arrest in G2/M phase Inhibition of CDK1 dephosphorylation	Zhang et al. (2019)
HeLa and SiHa cancer cells	Shikonin (0, 1.5, 2.5 and 3.5 µM	Induced downregulation of FAK, AKT phosphorylation Downregulation of GSK3β phosphorylation induced by EGF Reduced expression of migration-related proteins such as VEGF, MTA1, and TGFβ1	Xu et al. (2022)
Hela and C33a cells	Shikonin (0, 2, 4, 8, 16, 32 µM)	Inhibited EMT *via* snail inhibition and miR-183-5p stimulation Induction of E-cadherin expression	Tang et al. (2020)
HeLa and SiHa cells	Deoxyshikonin	Significantly triggered activation of p38 MAPK (p38), ERK, and JNK Only p38 inhibition diminished cleavage of DSK-mediated pro-caspases and PARP	Lee et al. (2024)
SiHa cervical cancer cell	Acetylshikonin (0, 0.025, 0.05, 0.25 μg)	Induced apoptotic cell death Altered DNA morphology in nucleus Cell cycle arrest at G2/M phase	Sun et al. (2014)

### 3.2 Anti-cancer potential of shikonin against ovarian carcinoma

Research has shown that the compound shikonin (SK), a type of naphthoquinone with anti-cancer properties, can reduce the production of tumor-associated exosomes (Meng et al., 2024). Shikonin achieves this by inhibiting exosome formation and blocking the activation of β-catenin mediated by exosomal GAL3, which in turn reduces the presence of M2 macrophages in ovarian cancer. Furthermore, shikonin was found to prevent the development of xenograft tumors in mice and limit the infiltration of M2 macrophages into tumor tissues (Wang et al., 2024). In advanced ovarian cancer, the overexpression of Src, a non-receptor tyrosine kinase, is notable, especially since inhibiting Src appears to overcome platinum resistance, partly by enhancing caspase-3-mediated apoptosis. As a result, various Src inhibitors are currently being tested, given Src’s potential as a target in ovarian cancer treatment (Manek et al., 2016). A study indicated that shikonin hindered ovarian cancer cell migration and cell death induction *via* blockage of two kinases phosphorylation including FAK and Src (Hao et al., 2015). Initial experiments revealed that shikonin was toxic to lymphocytes, normal ovarian IOSE-398 cells, ovarian epithelial cancer cells (OVCAR-5 and ID8 cells), and endothelial MS1 cells. To confine its cytotoxic efficacies to tumor cells within TME, SK was formulated as polymeric NPs with a specific component aimed at tumor microvasculature. The surface of these SK loaded NPs was further modified using carbodiimide/N-hydroxysuccinimide chemistry with PEG and a TME1/endosialin-targeting antibody. SK exhibited significant cytotoxic efficaciesagainst ovarian cancer cells. Consequently, a newly targeted nanomedicine for the treatment of ovarian cancer could be developed using biodegradable SK-loaded PLGA NPs that are PEGylated and equipped with anti-TEM1 scFv (Matthaiou et al., 2014).

A further investigation was designed to assess if shikonin could augment the anticancer efficacy of paclitaxel in human ovarian carcinoma cells that are resistant to drugs, considering its increasing significance in cancer treatment and in overcoming cancer multidrug resistance. The combination of shikonin and paclitaxel resulted in synergistic increase in cytotoxicity and cell death in paclitaxel-resistant ovarian cancer cells, validating that shikonin was able to bypass multidrug resistance associated with paclitaxel. However, in this context of ovarian carcinoma, multidrug resistance reversalby the shikonin/paclitaxel combination was achieved through a P-gp-independent mechanism involving ROS production (Hao et al., 2015).

Cisplatin-based chemotherapy is mainly used to treat ovarian cancer, but it often leads to the development of chemoresistance, which is difficult to overcome (Song et al., 2022; Ranasinghe et al., 2022). Shikonin induced cell death in shikonin treated ovarian cancer cells (SKOV3, A2780, A2780-CR, and SKOV3-PR) for 48 h. Shikonin triggers apoptosis in A2780-CR ovarian cancer cells through a mechanism dependent on mitochondria. Shikonin activated mitogen-activated protein kinases. This study suggests that shikonin aids A2780-CR cells in overcoming chemoresistance by promoting mitochondria-mediated apoptosis and inhibiting EMT (Shilnikova et al., 2018). Proteomic analysis also revealed that the combination of cisplatin and shikonin displayed ferroptosis process as evidenced by decreased glutathione peroxidase 4 (GPX4) and increased levels of Fe^2+^, ROS, and LPO. Combined impact of these two drugs on ovarian cell viability was reduced by siRNA interference and heme oxygenase 1 (HMOX1) inhibition. siRNA inhibition of HMOX1 led to a reduction in Fe^2+^accumulation. *In vivo*, the combination therapy augmented ferroptosis-related proteins expression in tumor tissue and significanttumor growth inhibition the tumor (subcutaneous) in BALB/c nude mice. Cisplatin resistance can be reversed by combined treatment of SK and cisplatinin ovarian cancer *via* triggering ferroptosis through HMOX1 overexpression, which enhances the accumulation of Fe^2+^ (Ni et al., 2023).

To ensure shikonin molecules are delivered accurately and effectively to cancer cells, as is necessary for all cytotoxic agents, nanoscale targeted drug delivery systems can be employed. Shikonin-loaded nanoparticles, equipped with antibodies, have been developed and validated as a potent targeted nanomedicine for treating ovarian carcinoma (Matthaiou et al., 2014). Even with chemotherapy, numerous patients suffer from considerable toxicity, frequently because of drug resistance and the unintended build-up of anticancer drugs in healthy cells or tissues. These negative effects can potentially result in the failure of treatment strategies (van den Boogaard et al., 2022). Advanced multifunctional nanomedicines with active and/or passive targeting capabilities have been successfully utilized to avoid the unintended side effects associated with traditional chemotherapy methods. It has been observed that smart targeted nanoparticles/nanosystems accumulate significantly in the tumor microenvironment through active targeting, which involves interaction with specific cancer antigens or molecular markers, and passive targeting, which utilizes increased retention effect and enhanced permeation (Pérez-Herrero and Fernández-Medarde, 2015; Ahmad et al., 2019; Arafat et al., 2024). This results in minimal side effects and maximized therapeutic effects in cancerous cells (62). Among anticancer nanosystems, the potential of biodegradable polymeric nanoparticles to deliver loaded anticancer drugs safely to target cells with minimal side effects has been extensively studied. PLGA is one of the most researched biocompatible polymers among biodegradable options and has been investigated for use as a delivery system (Perinelli et al., 2019).

In another investigation, PLGA nanoparticles (NPs) were modified with PEG, equipped with a segment of the anti-TEM1 antibody (78Fc), and infused with SK, which triggers necroptosis, resulting in 78Fc-PLGA-SK NPs. In aggressive tumor models such as TC1 murine lung carcinoma models (subcutaneous and intravenous/metastatic), this nanoformulation notably increased cytotoxicity. The 78Fc-PLGA-SK NPs were expelled through urine without accumulating in spleen or liver, yet their administration in MS1-xenograft mice led to a significant build-up and impact on TEM1-positive tumor targets (Matthaiou et al., 2021). A separate study evaluated the IC_50_ of shikonin and the growth curve for KURAMOCHI, OVSAHO, CP70, and ascites E04 cell lines. It also discovered that type 2 ovarian cancer cells underwent apoptosis due to activated apoptotic signaling pathway (Binju et al., 2019). This approach proved effective in treating type 2 ovarian carcinomas by decreasing the expression of the gene for type 2 ovarian cancer stem cells and reducing tumorigenicity of KURAMOCHI cell cancer stem cells by inducing apoptosis in NOD-SCID mice (Chang et al., 2022).

SK has demonstrated potential in treating ovarian carcinomavia ROS induction. Though, it has limited clinical usage due to its limited bioavailability and poor targeting of tumors, along with the high levels of GSH in tumor cells that diminish its effectiveness (Lu et al., 2024). To address this, ROS-responsive micelles formulation containing SK was developed using hyaluronic acid-phenylboronic acid pinacol ester conjugation (HA-PBAP) for targeted ovarian carcinoma therapeuticsvia disrupting the intracellular redox balance. SK@HA-PBAP formulation accumulated specifically in tumor tissues and concentrates in carcinoma cells *via* HA/CD44 receptor-mediated endocytosis. Upon encountering high ROS levels, SK@HA-PBAP disintegrates, releasing shikonin and quinone methide from the cancer cells. Quinone methide can deplete glutathione, while the released shikonin promotes ROS production. Ultimately, these processes resulted in a significant redox imbalance that efficiently eradicated tumor cells. Consequently, this ROS-responsive SK@HA-PBA formulation projects a promising viable therapeutic approach for ovarian carcinoma (Hu et al., 2024). Additionally, SK has been shown to effectively trigger apoptosis and proliferation inhibition in SKOV-3 cells. In both medium-dose and high-dose shikonin groups, there was a significant reduction in colony formation and cell survival rates (JIAO et al., 2024).

Another study explored how reducing PKM2 levels affects ovarian cancer cells sensitivity to PARPi that have shown therapeutic success in advanced ovarian carcinomavia inhibiting homologous recombination (HR) pathway (Gomez et al., 2020). PKM2 (key metabolic cancer marker) interacts with DNA damage to directly promote HR. PKM2 suppression using siRNA or the small molecule inhibitor SK increased the anti-cancer effects of olaparib (Ola) in ovarian cancer cells. PKM2 silencing or SK used in combination with Ola decreased cell proliferation, migration, and colony formation while inducing apoptosis. Inhibiting PKM2 disrupted the nuclear accumulation of BRCA1 and increased Ola-induced γH2AX and phospho-ATM (p-ATM) activation. The combined anticancer effects of SK and Ola were also observed *in vivo* using a xenograft animal model. Furthermore, SK treatment led to decreased expression of BRCA1 and PKM2 and increased DNA damage, as shown by Western blot and immunofluorescence analyses of tissue samples (Zhou et al., 2022). Table 2 summarized all significant studies associated with the mode of action of shikonin against cervical cell carcinoma.

**TABLE 2:** Anti-cancer potential of shikonin against ovarian cancer cells at different doses.

Cancer cell line (s)	Dose (s)	Mode of action	References
OC cells SKOV3 and A2780	Shikonin (1 μM, 2 μM, 4 μM, 8 μM, 16 μM, 32 μM, 64 μM, 128 μM, and 256 µM)	Reduced M2 macrophage population Repressed exosome production Exosomal galectin 3-mediated β-catenin activation Reduced tumorigenesis of SKOV3 cells in nude mice	Wang et al. (2019)
Ovarian carcinoma SKOV-3 cells	Shikonin (1, 2, 4, 8, 16, 32, 64, 128 or 256 μM)	Induces apoptosis Inhibited migration of ovarian carcinoma cells *via* inhibited Src and FAK phosphorylation	Hao et al. (2015)
Ovarian cancer SKOV3 and A2780 cells	Shikonin (0, 5, and 10 µM)	Apoptotic induction *via* suppressed GPER/EGFR/PI3K/AKT signaling pathway	Liu et al. (2021)
Human ovarian cancer cell lines (KURAMOCHI, OVSAHO, CP70, and Ascites E04)	KURAMOCHI (0.507 μM), OVSAHO (0.9165 μM), CP70 (0.258 μM), ascites E04 (1.338 μM)	Impeded type 2 ovarian cancer progression *via* modulated FAsL/caspase-8 and miR-874-3p/XIAP axis and in type 2 ovarian cancer cells	Chang et al. (2022)
Human ovarian cancer cell (SKOV-3) cells	Shikonin (0, 2.5, 5 and 10 μmol/L)	Reduced p-JNK1/2 and PCNA expression levels and increased caspases (9 and 3) expression levels *via* modulated JNK signaling pathway	JIAO et al. (2024)
Human ovarian cancer A2780, SKOV3 and OVCAR3 cell lines	Shikonin (4 μmol/L, 8 μmol/L and 6 μmol/L) Olaparib (1 μmol/L and 10 μmol/L)	PKM2 downregulation Enhanced anti-tumour activity of olaparib Defective homologous recombination pathway	Zhou et al. (2022)
Cisplatin-resistant human ovarian cancer A2780 cells (A2780-CR)	Shikonin (0.0, 2.5, 5.0, 10.0, 20.0, 30.0 or 40.0 µM)	Mitochondria-mediated apoptosis induction Attenuates epithelial-mesenchymal transition in cisplatin-resistant human ovarian cancer cells Induction of apoptosis by shikonin *via* MAPK activation	Shilnikova et al., 2018
A2780 cell along with the paired PTX-resistant A2780/PTX cells	Shikonin with paclitaxel (1, 10, 100 and 1,000 nM)	Multidrug resistance in human ovarian carcinoma cells in a P-gp-independent manner *via* enhanced ROS generation Downregulated PKM2	Wang et al. (2019)
Ovarian epithelial cancer cells (OVCAR-5 and ID8 cells)	Shikonon (15 mg/mL) and fluorescein (5 mg/mL) + 3 mL of oil phase (PLGA + surfactant)	Induced marked cytotoxic impacts Inadvertent detrimental impact on normal cells	Matthaiou et al. (2014)

### 3.3 Anti-cancer potential of shikonin against breast carcinoma

Subtypes of breast cancer (HER2, PR and ER) vary greatly in terms of occurrence, response to chemotherapy, drug resistance, tumor development, and patient survival (Parise et al., 2009; Abhilash et al., 2023). Advances in new BC chemotherapy methods have enhanced their anticancer effects, significantly boosting survival rates (Smolarz et al., 2022). However, these chemotherapeutic strategies still face major challenges, such as insufficient response to treatment and resistance to multiple drugs. Besides chemotherapy, hormone therapy is also utilised as non-targeted treatment for BC, but associated with severe limitations. Resistance to radiation can lead to cancer recurrence after treatment. Immunotherapy and targeted therapies aiming at specific targeting of malignant targets (biochemical) has emerged as a promising approach for BC (Stopeck et al., 2012; Kumari et al., 2023). Therefore, to address these limitations, it is crucial to identify newly developed strategies. Tumor cells can interact with and modify their environment by releasing exosomes, which are vesicles measuring 50–100 nm in diameter (Lowry et al., 2015; Kruger et al., 2014). These exosomes facilitate the transfer of nucleic acids, signals, lipids, and proteins, including microRNAs (miRNAs), from 1 cell to another. Modern research has linked exosomal miRNAs to tumor development, invasion, and progression. Shikonin (0–100 µM) treatment has been found to decrease miR-128 (tumor-derived exosome), thereby inhibiting MCF-7 cells proliferation. Donor Exosomes MCF-7 cells release miR-128 (exosomal) for getting absorbed by recipient MCF-7 cells. In these recipient cells, miR-128 cells can downregulate Bax gene and promote cell division. By reducing exosome secretion, shikonin treatment can thus hinder MCF-7 cell growth. This study demonstrates that SK suppresses MCF-7 cells growth by decreasing tumor-derived exosomes (Wei et al., 2016).

Most individuals with breast cancer display the estrogen receptor (ER) (Haldosén et al., 2014; Duffy, 2006). Metastasis and progression of breast cancer tumors are associated with the membrane-bound ER known as the GP (G protein-coupled) ER (Hsu et al., 2019; Girgert et al., 2019). A study investigated whether the ER/GPER signaling pathway is accountable for shikonin’s ability to trigger apoptosis. Shikonin treatment inhibited MCF-7 cells by inducing apoptosis and cell growth arrest (G0/G1) phase. MCF-7 cells expressed both GPER and ERα, while SK-BR-3 cells were positive for GPER and negative for ERα. Shikonin also reduced expression levels of EGFR and p-ERK in both SK-BR-3 and MCF-7. Downregulation of EGFR/p-ERK through the suppression of GPER and ERα seems to be related to these effects (Yang et al., 2019). The enzyme steroid sulfatase (STS) is crucial in regulating estrogen production in breast cancers. SK altered STS expression in BC cells by blockingMCF-7 and SK-BR-3 cell proliferation. Additionally, STS’s mRNA and enzymatic activity levels were also reduced after shikonin treatment. Thus, by inhibiting STS expression, shikonin may act as a selective regulator of estrogen enzymes. Shikonin (0–80 μM) treatment was given to MCF-7 and SK-BR-3 cells (Zhang et al., 2009). BC in mice derived from BALB/c 4T1 cells was selected for further research due to their growth characteristics and systemic response, which closely resemble those of human breast cancer. Shikonin may inhibit cell division, induce apoptosis, disrupt mitochondrial function, and lead to ROS generation and CRT exposure *in vitro* (4T1 cells). *In vivo*, shikonin reduced the percentage of regulatory cells (CD^25+^ Foxp^3+^ T cells) in spleen, increased the percentage of CD^8+^ T cells, and inhibited tumor growth. Through various mechanisms, such as relieving immune suppression, enhancing oxidative stress, and disrupting mitochondrial activity, SK can halt 4T1 BC growth. This study helped inidentifying the optimal dosage and potential limitations of shikonin *in vivo* for treating breast tumors (Yu et al., 2024).

Inosine 5′-monophosphate dehydrogenase 2 (IMPDH2) is an enzyme that regulate the speed of *de novo* guanine nucleotide synthesis and therefore considered a potential target for cancer therapeutics due to its consistent overexpression in various TNBCs (Fotie, 2018). Enzymatic studies using the Lineweaver-Burk plot have shown that SK acts as competitive inhibitor of IMPDH2. Shikonin treatment effectively curtails the proliferation of the human TNBC cell line MDA-MB-231 and the murine TNBC cell line 4T1 (dose-dependent manner), although this effect is mitigated by the addition of exogenous guanosine, a component of the purine nucleotide salvage pathway. The overall findings of the research suggest that shikonin is a specific inhibitor of IMPDH2 with anti-TNBC properties, warranting further clinical trials (Wang A. et al., 2021). In breast cancer tissues, there is an abnormal overexpression of PDK1 (key enzyme in the glucose metabolism pathway) promote tumor growth and metastasis (Du et al., 2016). PDHC/PDK axis and PDK1 are significant targets for regulatingglucose metabolism and anti-tumor and activity. Several semi-synthesized shikonin (SK) derivatives were evaluated for their anti-tumor effects on human BC cells. The findings indicated that SK derivatives significantly blocked the growth of MDA-MB-231 cells. E2 and E5 (novel SK derivatives) disrupted tumor glycolysis and induced apoptosis by specifically targeting PDK1. Consequently, E2 emerges as a promising lead drug (new PDK1 inhibitor) used for TNBC treatment (Chen et al., 2022).

RNA-seq transcriptome analysis was employed to explore how shikonin affects cell proliferation of different BC cells. Shikonin induced apoptosis in MDA-MB-231 cells and halted progression of cell cycle. It also increased the RNA and protein levels of dual specificity phosphatase (DUSP1 and DUSP2). Additionally, shikonin inhibited p38 and JNK, which are downstream signaling molecules of DUSP1 and DUSP2. These findings suggested cell cycle arrest and apoptosis induction in SK treated BC cells *via* upregulated DUSP1 and DUSP2, thereby inhibiting the p38/MAPK/JNK pathways (Lin K. H. et al., 2018). *In vitro*, shikonin triggered p38-dependent apoptosis in both MDA-MB-231 human BC cells and murine mammary cancer cells, resulting in anti-tumor effect. The anti-tumor effects of shikonin were also examined in orthotopic mouse models. Tumor volumes in SK treated group began to differ from the control group on the seventh day after 4T1 cells were injected into mice. By day 13, SK suppressed tumor growth in the orthotopic 4T1 model. Shikonin emerged as an anti-tumor agent for BC cells, including MDA-MB-231 cells and 4T1 murine mammary carcinoma (Xu et al., 2019). Further studies investigated shikonin’s impact on invasion and migration of human BC. Shikonin inhibited MMP-9 production and its proteolytic and promoter activity, thereby preventing phorbol 12-myristate 13-acetate (PMA) from inducing cell invasion in MCF-7 BC cells. Moreover, shikonin reduced MMP-9 promoter activation in MDA-MB-231 cells. These findings indicate that SK inhibited migration and progression in human BC cells by modulating MMP-9. Thus, SK may be a promising anti breast cancer drug (Jang et al., 2014).

**FIGURE 2:**
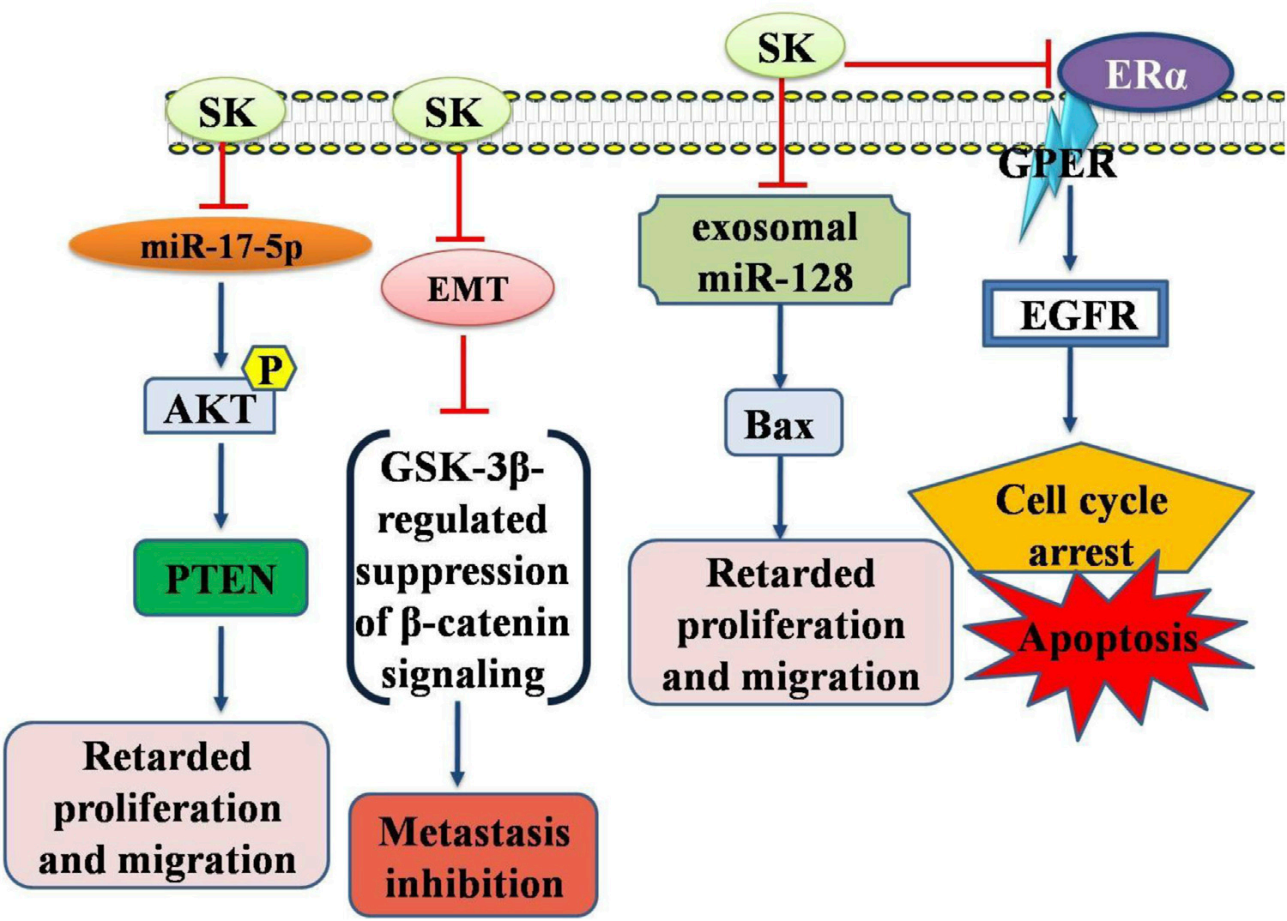
Diagrammatic illustration of the anti-breast cancer potential of shikonin. Shikonin has been shown its inhibitory or promoting effects on certain genes or proteins required either for the cancer cell death or inhibition of the genes required for apoptosis in cancer cells. Abbreviations: EGFR, Epidermal growth factor receptor; AKT, Protein Kinase B (PKB); PTEN, Phosphatase and TENsin homolog deleted on chromosome 10.

EMT is regarded as the most detrimental phase in metastasis, and thus pharmacologically targeting EMT could be a viable strategy to enhance the therapeutic effectiveness of TNBC (Neophytou et al., 2018; Li et al., 2018). Moreover, shikonin has demonstrated considerable success in inhibiting EMT. By suppressing glycogen synthase kinase 3β mediated β-catenin signaling, SK reversed EMT transition and hindered the metastasis of TNBC. Shikonin altered arrangement of cytoskeletal proteins (F-actin and vimentin), reduced cell migration, elevated E-cadherin levels, and lowered levels of N-cadherin and Snail. Histological analysis revealed that shikonin decreased vimentin and β-catenin levels in lung metastatic sites while increasing GSK-3β, E-cadherin, and phosphorylated β-catenin. These findings underscore SK potential as promising candidate for novel anti TNBC therapies, as it effectively inhibits TNBC metastasis viaEMT targeting through reduction of β-catenin signaling (GSK-3β-regulated) (Chen et al., 2019). Similarly, another study examined SK impact on EMT. LPS enhanced cell motility and invasion by inducing phenotypic changes akin to EMT. SK significantly increased expression levels of E-cadherin in MCF-7 cells and reduced LPS-induced EMT markers expression such as N-cadherin in MDA-MB-231 cells. *In vitro*, SK also inhibited cell invasion and migration. SK mediated its effects on LPS-induced EMT *via* inactivated NF-κB-Snail signaling pathway. These findings provided new evidence aboutSKmediated EMT inhibition to prevent BC cells from invading and migrating. Therefore, SK may serve as an effective anticancer treatment for BC by preventing metastases (Hong et al., 2015). Moreover, SK was found to impede the migration in BT549 and MDA-MB-231 cells. Concurrently, SK treated MDA-MB-231 cells exhibited similar alterations in EMT biomarkers. Shikonin also reduced the miR-17-5p expression, which is typically elevated in BC. EMT and metastasis of TNBC cells were inhibited by PTEN. Additionally, shikonin hindered EMT and migration of BC cells by engaging Akt and p-Akt (Ser473). SK effectively suppressed EMT viamiR-17-5p/PTEN/Akt pathway thereby preventing TNBC cell migration (Bao et al., 2021) (Figure 2).

Targeted therapeutic drugs can eliminate cancer cells that resist apoptosis by utilizing necrotic signaling pathways (Carneiro and El-Deiry., 2020). To evaluate shikonin’s effect on inducing necroptosis or apoptosis, the T-47D breast cancer cell line was employed. Necroptosis was identified as the main mechanism of cell death. Shikonin treated cells in the presence of Nec-1 exhibited caspase-3-mediated apoptosis. Shikonin facilitates necroptosis or apoptosis by triggering ROS production in T-47D mitochondrial cells. Inducing necroptosis, a secondary programmed cell death process activated by ROS, offers a new strategy for breast cancer treatment (Shahsavari et al., 2015). In a similar study, SK effect on RIPK1-RIPK3-mediated necroptosis and apoptosis was examined in MCF-7 (ER + breast cancer) cells. SK induced both necroptosis and apoptosis, with a notable increase in expression levels of both RIPK1 and RIPK3. However, necroptosis was dominant pathway in MCF-7 cells. SK significantly increased cell growth arrest at the sub-G1 and later cell cycle stages, indicating increased necroptosis and apoptosis. Nec-1 prevented shikonin-induced necroptosis when Z-VAD-FMK inhibited caspase which ultimately resulted in reducedMMP and enhanced ROS levels (Shahsavari et al., 2018). Furthermore, SK significantly induced apoptosis and necrosis in MDA-MB-231 cells by enhancing autoubiquitination levels and promoting the proteasome-dependent degradation of cellular inhibitor of apoptosis protein 1 (cIAP1 and cIAP2). SKinduced degradation of cIAP1 and cIAP2 proteins and autoubiquitination led to a significant decrease in RIP1 inactivation and ubiquitination that played a vital role in inhibiting pro-survival pathways and augmenting necrosis in MDA-MB-231 cells. Consequently, shikonin could potentially be further investigated as novel therapeutic option for TNBC treatment (Wang W. et al., 2021).

In cancerous cells, resistance to cell death and metabolic reprogramming are essential factors. The primary challenges in effectively treating triple negative breast cancer are heightened resistance to apoptosis and tumor recurrence. It is thought that ROS production and mitochondrial dysfunction contribute to necroptosis process in cancerous cells (Hsu et al., 2020; Gong et al., 2019). SK treated MDA-MB-468 cells exhibitedreduced MMP and an increased ROS levels. Recent studies suggest that SK induces augmented ROS levels in the mitochondria of the TNBS cells, which can act as double-edged sword in the context of apoptosis or necroptosis. SK primarily induces cell death through RIP1K-RIP3K-mediated necroptosis; however, in the presence of Nec-1, apoptosis becomes predominant. These findings offer new perspectives on treating drug-resistant TNBC (Shahsavari et al., 2016). Table 3 summarized all significant studies associated with the mode of action of shikonin against breast carcinoma. One clinical trial (NCT01287468) has been reported against breast cancer includes “Academia Cinica investigator award” by shikonin in the year 2010.

**TABLE 3:** Anti-cancer potential of shikonin against breast carcinoma at different doses.

Cancer cell line (s)	Dose (s)	Mode of action	References
MCF-7 cells	Shikonin (0–100 µM)	Proliferation inhibition using reduced tumor-derived exosomal miR-128 Exosomal miR-128 downregulated Bax gene	Wei et al. (2016)
MCF-7 and SK-BR-3 cells	Shikonin (1 × 10^−5^ mol/L, 1 × 10^−6^ mol/L, 1 × 10^−7^ mol/L and 10^−8^ mol/L)	G0/G1 arrest and apoptosis induction EGFR/p-ERK downregulation *via* ERα and GPER inhibition	Yang et al. (2019)
MCF-7 and SK-BR3 cells	shikonin (0.625, 1.25, 2.5, 5, 10, 20, 40, 80 μM)	Reduced mRNA and enzymatic activity levels Selective estrogen enzyme modulator *via* downregulated steroid sulfatase expression level	Zhang et al. (2009)
BALB/c-derived mouse breast cancer 4T1 (*In vivo*)	Shikonin ranging from 10 to 0.039 μg/mL	Tumor growth inhibition Enhanced proportion of CD8+ T cells Reduced proportion of regulatory cells (CD25^+^ Foxp3^+^ T cells) in the spleen Disruption of mitochondrial activity Promotion of oxidative stress Relief of immune suppression	Yu et al. (2024)
4T1 cells	Shikonin ranging from 10 to 0.039 μg/mL	Similar to DOX, shikonin may kill tumor cells by inducing increased ROS production Inhibition of 4T1 breast cancer cell proliferation *In vitro* Attacks and disrupts mitochondrial functioning of 4T1 cells	Yu et al. (2024)
MDA-MB-231 and 4T1 cells	Shikonin (0.5 or 5 μM)	Emerged as novel and specific IMPDH2 inhibitor Competitive and reversible IMPDH2 inhibitor	Wang A. et al. (2021)
MCF-7, SKBR-3 and MDA-MB-231 cells	Shikonin (10, 20, 30, 40 and 50 μM)	Arrested cancer cell progression in G1 phase of the cell cycle Induced apoptosis Enhanced expression of DUSP1 and DUSP2 in both RNA and protein levels Decreased phosphorylation of JNK and p38	Lin K. H. et al. (2018)
4T1 murine mammary cancer and MDA-MB-231 cells (*In vitro*)	shikonin (0–8 µM)	Inhibited cell proliferation Caspase-3/7 and p38 signaling pathway dependent apoptosis induction	Xu et al. (2019)
Female BALB/c mice (6 weeks old) (In vivo)	Shikonin (2 mg/kg)	Suppressed orthotopic 4T1 tumor growth	Xu et al. (2019)
MDA-MB-231 and MCF-7 cells	Shikonin (0.2, 0.5, 1, 2, 5, 10, 20 and 50 µM)	Reduced migration of cancer cells during LPS induced EMT Suppressed LPS-triggered EMT *via* counteracting LPS induced NF-κB p65 activation and snail induction	Jang et al. (2014)
MDA-MB-231 and 4T1 cells	Shikonin (0.2, 0.4, and 0.8 µM)	Inhibited β-catenin signaling *via* increasing GSK-3β levels Suppressed β-catenin expression and nuclear translocation Inhibited lung metastasis and β-catenin signaling in NOD/SCID mice inoculated with MDA-MB-231 cells	Chen et al. (2019)
MDA-MB-231 and MCF-7 cells	Shikonin (0.2, 0.5, 1, 2, 5, 10, 20 and 50 µM)	Reduced migration of cancer cells during LPS induced EMT Exert an inhibitory effect on EMT Inactivation of NF-κB-Snail signaling pathway	Hong et al. (2015)
MDA-MB-231 and BT549 cells	Shikonin (0.5, 1, 2.5, and 5 µM)	Inhibited overexpressed miR-17-5p expression levels Suppressed EMT *via* miR-17-5p/PTEN/Akt pathway	Bao et al. (2021)
T-47D cells	Shikonin (0.5, 2.5, 5, 10, 15, 20, 25 µM)	Inhibited cell proliferation and induces cell death Caspase-8,3 activation and mediate d ROS production	Shahsavari et al. (2015)
MDA-MB-231 cells	Shikonin (0.625, 1.25, 2.5 and 5 µM	SKO induced cytotoxicity was independent of activated RIP1/RIP3/MLKL axis Facilitated ubiquitination of cIAP1 and cIAP2 Induced degradation of RIP1, cIAP1 and cIAP2 Increased auto-secretion of TNF-α for apoptosis and necrosis induction	Wang W. et al. (2021)
MDA-MB-468 cells	Shikonin (0.5, 2.5, 5, 10, 15, 20 25 μM)	Increased ROS levels and reduced mitochondrial membrane potential Cell death *via* necroptosis with a significant increase in RIP1K and RIP3K expression levels	Shahsavari et al. (2016)

**FIGURE 3:**
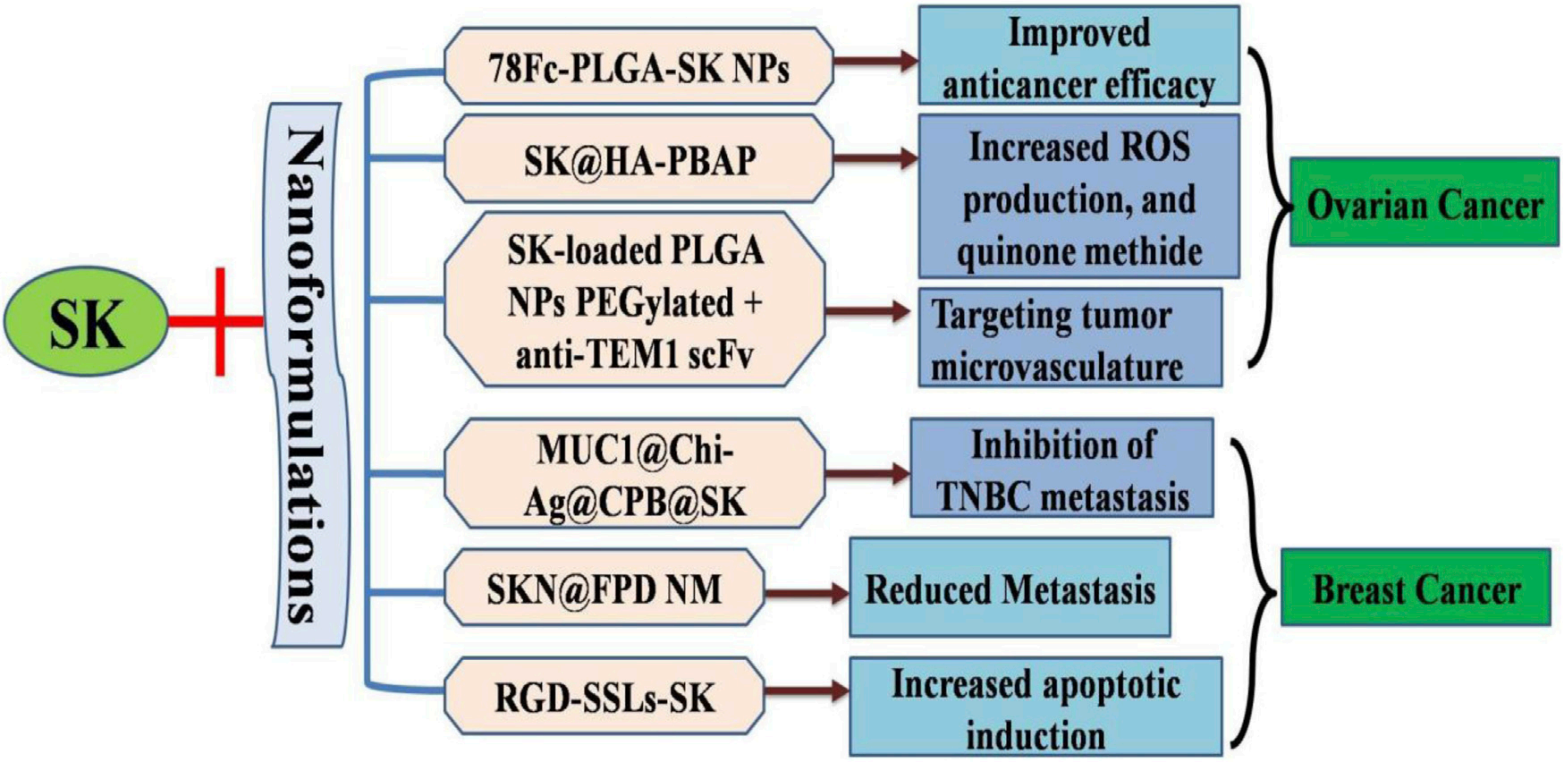
Diagrammatic illustration of nanoformulatios of shikonin to increase targeted drug delivery and increased apoptotic efficiency.

Triple-negative breast cancer (TNBC) metastases and recurrences can be addressed by activating the human immune system through necrotic immunogenic cell death (ICD) (Kim and Kin., 2021; Cheng et al., 2023). However, the primary challenge lies in developing a necroptotic inducer and its accurate delivering to the tumor site. Significant necroptosis mediated ICD was observed in 4T1 cells that were treated with SK and chitosan silver nanoparticles (Chi-Ag NPs). A MUC1 aptamer-targeted nanocomplex, known as MUC1@Chi-Ag@CPB@SK or MUC1@ACS, was designed to co-deliver SK and Chi-Ag NPs. MUC1@ACS NPs accumulation at the tumor site was 6.02 times greater than that of free drug. At tumor site, MUC1@ACS NPs released SK and Chi-Ag NPs in response to acidic environment, causing tumor cell necrosis by increasing expression levels of tetrameric MLKL, RIPK3, p-RIPK3, and, which subsequently triggered ICD. This process led to Treg cells inhibition and enhanced infiltration of T cells (both CD8^+^ and CD4^+^) within tumors, effectively preventing TNBC metastasis *via* treating both primary tumor and distant tumor growth (Liang et al., 2024). These findings highlighted the vital role of nanoparticles in facilitating drug to target interactions during necroptotic ICD (Figure 3).

The widespread application of this compound in clinical settings is limited due to its inadequate water solubility and lower bioavailability. In this research, RGD-modified shikonin-loaded liposomes (RGD-SSLs-SK) were successfully developed to address these challenges. These liposomes exhibited remarkable physicochemical characteristics, such asextended release time, particle size, encapsulation efficiency, and zeta potential (Wen et al., 2018). It is evident that RGD-SSLs-SK can induce apoptosis *via* modulating Bax (increase) and Bcl-2 (decrease) expression levels. Additionally, it may inhibit cell migration, adhesion, and proliferation *via* reducing NF-κB p65 and MMP-9 expression levels, although it did not affect MMP-2 expression in MDA-MB-231 BC cells. Consequently, these findings suggested the usage of RGD-modified liposomes as carriers for targeted SK delivery and projected a highly viable approach for targeted breast cancer therapy. The results demonstrated that free SK, RGD-SSLs-SK, and shikonin-loaded liposomes (SSLs-SK) all have the ability to reduce cellular growth sand free SK could swiftly penetrate the cytomembrane and enter the intracellular space, whereas the liposomes were internalized through endocytosis after a certain period (90).

In recent years, chemoimmunotherapy has proven to be an effective cancer treatment, yet TNBC remains without definitive cure (Jia et al., 2017; Liu et al., 2023). Theseunsatisfactoryfindings are probably due to insufficient tumor immunogenicity and tumor metastasis (immunosuppressive TME). To overcome these challenges, a successful TNBC chemoimmunotherapy was developed by integrating an efficient delivery system with siTGF-β, SK, and TGF-β. The SK/siTGF-β nanoparticles (NPs) (approximately 110 nm) demonstrated good stability and a uniform structure. Silencing TGF-β reduced inhibited EMT and TGF-β-mediated ITM, which contributed to limiting Treg proliferation, enhancing CTL infiltration, and preventing lung metastasis. Consequently, SK/siTGF-β NPs exhibited highest therapeutic efficacy by delaying tumor progression and restraining lung metastasis. Additionally, the codelivery approach blocked tumor recurrence by inducing a long-lasting immune memory response. SK/siTGF-β NPs, which focus on boosting IR and inhibiting the ITM, present a potential strategy for TNBC treatment (Li J. et al., 2022). A significant challenge in breast cancer endocrine therapy is tamoxifen resistance. LNC RNAs plays vital role in tumor growth. Compared to the original MCF-7 cells, tamoxifen-resistant MCF-7R cells showed increased BCL11A (mRNA and protein) expression levels and decreased uc.57 levels. Moreover, breast cancer tissues exhibited higher BCL11A mRNA and lower uc.57 mRNA levels compared to precancerous breast cells. SK treatment reduced tamoxifen resistance in MCF-7R cells *via* targeting uc.57/BCL11A. Overexpressed Uc.57 levels reduced tamoxifen resistance and downregulated BCL11A in MCF-7R cells. Furthermore, knocking down BCL11A decreased tamoxifen resistance *via* blockingPI3K/AKT/MAPK cell signaling pathway. Thus, it appears that SK reduces tamoxifen resistance in MCF-7R BC cells *via* activating uc.57, which suppressedPI3K/AKT/MAPK signaling pathways *via* BCL11A downregulation (Zhang et al., 2017).

The combined action of metformin intensified the significant reduction in cell viability caused by shikonin. This drug pairing completely halted cell migration, reversed epithelial-mesenchymal transition (EMT), and triggered both apoptosis and increased ROS levels. Augmented BAX and PTEN expression and reduced BCL-2 expression levels were shown by *in vitro* experimentation. Metformin encouraged apoptotic cell morphology and mitigated damage, whereas extended exposure to shikonin led to the total disintegration of the nuclear membrane. Real-time PCR analysis indicated an upregulation of the anti-EMT gene CDH1, while EMT gene levels were suppressed. Furthermore, the combination therapy reduced CD44/CD24 ratios, enhancing chemosensitivity. Shikonin’s interactions with growth-signaling molecules were significantly enhanced by binding energies. Together, shikonin and metformin inhibit the tumorigenic characteristics of MCF-7 cells, such as proliferation, invasion, and EMT, and they may also help prevent multidrug resistance (Tabari et al., 2022). The creation, synthesis, and nuclear magnetic resonance analysis of a novel copolymer with adjustable block lengths of poly (N-isopropylacrylamide) and polylactic acid have been accomplished. Subsequently, a thermosensitive nanomicelle (TN) with a unique core-shell configuration is assembled in an aqueous solution. A shikonin-loaded thermosensitive nanomicelle (STN) is formed by incorporating shikonin into a biodegradable inner core. Shikonin specifically triggers programmed cell death (PCD), enhancing the therapeutic impact. Notably, PCD and the inhibition of proliferation synergize when T > VPTT (temperature-regulated passive targeting). Consequently, STNs accumulation within tumors is significantly augmented when T > VPTT during intravenous administration to BALB/c nude mice with BC, further validating improved synergist therapeutic effectiveness. Thus, STN could be an effective nanoformulation for clinical cancer therapy (Su et al., 2017).

Research has shown enhanced oxidative stress is effective against cancer (Klaunig, 2018; Hayes et al., 2020). SK helps regulate oxidative stress. To treat TNBC, hyaluronic acid-coated shikonin liposomes (HA-SK-Lip) were developed. These liposomes exhibited slow drug release and high drug encapsulation efficiency. Studies on anticancer properties of HA-SK-Lip revealed that they significantly blocked MDA-MB-231 cells growth. HA-SK-Lip uptake *via* CD44 receptor-mediated endocytosis pathway in BC cells was significantly elevated compared to SK-Lip and frees SK. Further analysis indicated that HA-SHK-Lip could significantly reduce intracellular glutathione (GSH) levels and enhance ROS production. In BALB/C mice with MDA-MB-231 tumors, anticancer efficacy of HA-SK-Lip was notably superior to that of free SK and SK-Lip. These results suggest that HA-SK-Lip mediated targeting MDA-MB-231 tumor cells and augmenting cellular oxidative stress could be a strong therapeutic approach for TNBC management (Meng et al., 2022).

One potential target for treating TNBC is the mitochondria (Weiner-Gorzel and Murphy., 2021). SK targets polymerase gamma (POLG) to exert strong inhibitory potential on mitochondrial biogenesis. Due to SK’s poor water solubility and stability, biomimetic micelle is designed to improve the poor water solubility thereby facilitatingmitochondria-targeted delivery and tumor lesion formation. To create a “right-side-out” RBCm-camouflaged cationic micelle (ThTM/SK@FP-RBCm), a folic acid (FA) conjugated polyethylene glycol derivative (FA-PEG-FA) is applied to outer membranes of red blood cells (FP-RBCm). Triphenylphosphine (TPP) moiety and FP-RBCm coating on the micelle’s surface enhance tumor lesion distribution, electrostatic attraction-dependent mitochondrial targeting, and receptor-mediated cellular uptake thereby enhancing the inhibitory potential on mitochondrial biosynthesis in TNBC cells. Administered intravenously, ThTM/SK@FP-RBCm significantly reduces lung metastasis and tumor growth in a TNBC animal model without noticeable harm. These outcomes displayed suppressed mitochondrial biogenesis and improved therapeutic outcomes for TNBC (Peng et al., 2022). Mammaglobin-modified liposomes loaded with shikonin (MGB-SK-LPs) are designed for targeted breast cancer treatment. The MGB-SK-LPs developed in this study can selectively target breast cancer cells, concentrate drugs on the tumor cell surface, and release them gradually. They also have the potential to significantly enhance SK’s anticancer therapeutic efficacy *in vivo*, providing a promising option for targeted breast cancer therapy (Zhang et al., 2022).

Polypeptide nanogel (effective against tumor microenvironment) holds significant promise as an effective anticancer treatment (Liu et al., 2021; Li Z. et al., 2022). A one-step ring-opening polymerization process was employed to synthesize a GSH responsive methylated PEG-poly (phenylalanine)-poly (cystine) block copolymer (mPPC). SK, referred to as mPPC/SHK, was encapsulated within the nanogel. Due to its enhanced permeability, retention effects and biocompatibility, mPPC accumulate at the tumor site, leading to the efficient release of SK when triggered by elevated GSH level. GSH responsive polypeptide nanogel encapsulating SK shows effective potential as tumor nanotherapy (Li et al., 2025). TNBC which often has poor prognosis is susceptible to metastasis and drug resistance. SK inhibits the epithelial-mesenchymal transition (EMT) pathway, which is typically linked to the significant activation of these TNBC characteristics. To load the SK, a folic acid-linked PEG nanomicelle (NM) was created and combined with DOX (referred to as FPD). They developed the SK@FPD NM using the drug loadings of DOX and SK and their dual drug effective ratio. Consequently, the combination of SK and doxorubicin (DOX) is expected to enhance anti-tumor activity and reduce metastasis (Zhong et al., 2023).

## 4 Efficacy of shikonin against endometrial cancer

Shikonin plant exhibits anticancer effects against numerous cancers. It is aneffective therapeutic agent for endometrioid endometrial cancer with its significant antiproliferative and apoptosis-inducing effects *via* modulating the miR-106b/phosphatase and tensin homolog/AKT/mTOR signaling pathway (Huang and Hu., 2018). Endometriosis, a common condition in women of reproductive age, is characterized by the infiltration of mononuclear cells into lesions (Ahn et al., 2015). Shikonin has been shown to inhibit the progression of endometriosis through several mechanisms, including reduced migration of mononuclear cells to lesions and reduced RANTES (chemokine for mononuclear macrophages) expression. In mouse/human chimera models, shikonin may prevent the growth of ectopic endometrial tissue. Its therapeutic effects might be attributed to the reduction of peritoneal inflammation, downregulated RANTES expression and inhibited monocyte recruitment in the peritoneal cavity of females with endometriosis. Further investigation into this compound could lay the groundwork for developing new treatments for endometriosis (Yuan et al., 2014). Altogether, more clinical investigations are needed to be done to elucidate potent treatment for female cancers.

## 5 Conclusion and future perspective

Shikonin (prominent bioactive substance) found in Lithospermum erythrorhizon, has been recognized for its ability to effectively destroy numerous carcinomas. Its antitumor properties target multiple pathways and mechanisms, attracting considerable interest and research in recent years. This review reports all possible advancements in elucidating the anticancer potential of SK and its nanoformulations *via* emphasizing its effect on different cell signaling pathways. This encompasses boosting ROS production, inhibiting angiogenesis, and triggering necroptosis and apoptosis. In summary, our review underscores the potential of shikonin and proposes strategies for employing it and its nanoformulations in the effective treatment of cancer. Due to much unlimited data, potential clinical translation and research are need in this direction. However, more *in vivo* and potential clinical translation and clinical studies are needed to validate its candidature to be utilized as anti female cancer lead candidate.

## Glossary

SK: Shikonin
ERK: Extracellular Signal-Regulated Kinas
ROS: Reactive oxygen species
PKM2: Pyruvate kinase
EMT: Epithelial-mesenchymal transition
β-HIVS: β-hydroxyisovaleryl-shikonin
GAL3: Galectin-3
FAK: Focal Adhesion Kinase
GPX4: Glutathione peroxidase 4
HMOX1: Heme oxygenase 1
HA-PBAP: hyaluronic acid-phenylboronic acid pinacol ester conjugation
PKM2: Pyruvate Kinase M2
HR: Homologous recombination
Ola: Olaparib
BRCA1: Breast Cancer 1, Early Onset
STS: Steroid sulfatase
CRT: Calreticulin protein
IMPDH2: 5′-monophosphate dehydrogenase 2
PDK1: 3-phosphoinositide-dependent protein kinase 1
PTEN: Phosphatase and tensin homolog
ICD: Immunogenic cell death (ICD)
TGF-β: Transforming growth factor-beta
STN: Shikonin-loaded thermosensitive nanomicelle
TNBC: Triple-Negative Breast Cancer
POLG: Polymerase gamma
TPP: Triphenylphosphine
MGB-SK-LPs: Mammaglobin-modified liposomes loaded with shikonin
DOX: Doxorubicin
DUSP: Dual specificity phosphatase
